# Ensuring Interrater Reliability When Evaluating Voice Assistants. Comment on “Evaluating Voice Assistants’ Responses to COVID-19 Vaccination in Portuguese: Quality Assessment”

**DOI:** 10.2196/36610

**Published:** 2022-03-21

**Authors:** Rujittika Mungmunpuntipantip, Viroj Wiwanitkit

**Affiliations:** 1 Private Academic Consultant Bangkok Thailand; 2 Department of Community Medicine DY Patil University Pune Thailand

**Keywords:** voice assistant, natural user interface, Portuguese language, COVID-19, vaccine

We would like to share our ideas on the paper “Evaluating Voice Assistants’ Responses to COVID-19 Vaccination in Portuguese: Quality Assessment” [1]. Seródio Figueiredo et al [1] concluded, “Under the urgent context of COVID-19 vaccination, this work can help to understand how VAs must be improved to be more useful to the society and how careful people must be when considering VAs as a source of health information.” We agree that voice assistants (VAs) could be useful in managing COVID-19 mass immunization campaigns. Current reports can provide an overview of existing technologies and the mission of various VA suppliers in order to meet current information distribution requirements. However, as previously said, the question of system functioning remains critical. Findings on VAs still have variability and need to be harmonized [2]. In addition, effective governance is required for transparency and usefulness in information partnerships [2].

Seródio Figueiredo et al [1] assessed VAs via 2 evaluators. A set of questions was used. The focus was on agreement between the 2 evaluators. Interevaluator variability should be assessed and presented as well. In general, there should be 3 evaluators to aid the final decision. Additionally, the scoring system of this study might be easily influenced by bias. Essentially, any questionnaire-based study requires a reliability test of the questionnaire. Content validity, face validity, and criterion-related validity tests are required. A standard method, as presented by Bolarinwa [3], should be used. A statistical analysis of the questionnaire’s reliability must be presented in addition to a general description. Without proven measures of questionnaire reliability and evaluator variability, the use of this tool in this study might be questionable.

## Abbreviations

VA: voice assistant
